# A Twice-Vanishing Lesion: Recurrent Stroke Mimic Reveals Cerebral Amyloid Angiopathy-Related Inflammation

**DOI:** 10.7759/cureus.92546

**Published:** 2025-09-17

**Authors:** Faizah Lubna, Joshua Martin Manogaran, Akash Jha, Monika Jha, Jawairia Fahim

**Affiliations:** 1 Internal Medicine, The Hillingdon Hospital NHS Trust, London, GBR; 2 General Internal Medicine, The Hillingdon Hospital NHS Trust, London, GBR; 3 Medicine, The Hillingdon Hospital NHS Trust, London, GBR; 4 Internal Medicine, Broomfield Hospital, Chelmsford, GBR

**Keywords:** adult neurology, angiopathy, brain tumors cns tumors, caari, cerebral amyloid angiopathy (caa), elderly population, pulse dose steroids, stroke, stroke mimic

## Abstract

Cerebral amyloid angiopathy-related inflammation (CAA-RI) is an uncommon inflammatory complication of CAA that can mimic stroke. Early recognition is critical, as the condition is often responsive to immunosuppressive therapy. We report a case of CAA-RI in a 63-year-old woman who presented twice with stroke-like symptoms, recurring seven years after the initial episode. Diagnosis is made on the basis of clinical suspicion and characteristic brain findings, which can be reversed with or without steroids. She first presented with confusion and vertigo. Magnetic resonance imaging (MRI) of the head with contrast showed increased T2 and fluid-attenuated inversion recovery (FLAIR) signal, along with hyperintensity on diffusion-weighted imaging (DWI) and apparent diffusion coefficient (ADC) in the right temporal lobe. She was started on dexamethasone 8 mg twice daily for a suspected tumor. On further review of the scans in the neuroradiology multidisciplinary team (MDT) meeting, the pattern of enhancement was not typical of a tumor, and the presence of punctate areas of hemosiderin deposition in both cerebral hemispheres on gradient-echo T2-weighted axial images was more suggestive of cerebral amyloid angiopathy/vasculopathy. Two months later, there was complete clinical and radiological resolution. After seven years, she presented with bilateral arm numbness and aphasia lasting for one hour. MRI showed features of a subacute infarct in the left middle cerebral artery territory, and the patient was treated with high-dose aspirin in view of the stroke. MRI performed for further characterization demonstrated subcortical T2 hyperintensity in the left temporoparietal region, with no underlying mass effect and no enhancement on post-gadolinium T1-weighted or FLAIR images. These findings were suggestive of cortical epileptic changes. The presence of subarachnoid FLAIR high signal was also noted, raising the possibility of meningitis or cerebritis. This was ruled out with a lumbar puncture (LP). Following a multidisciplinary team (MDT) discussion, she was diagnosed with cerebral amyloid-related seizure disorder. Repeat MRI at two months showed significant resolution of radiological changes, and MRI of the head after one year demonstrated complete radiological resolution. The diagnostic challenge faced by the treating physician, due to the clinical dilemma arising from varied presentations, can often lead to delayed or missed diagnosis. However, prompt recognition and treatment may result in full clinical and radiological recovery.

## Introduction

Cerebral amyloid angiopathy-related inflammation (CAA-RI) is an uncommon but increasingly recognized inflammatory response associated with amyloid-beta deposition in cerebral blood vessels [1,2]. It represents an aggressive yet often reversible subtype of cerebral amyloid angiopathy (CAA), distinguished by both characteristic amyloid deposits and additional inflammatory infiltration [3,4]. Two distinct pathological subtypes of CAA-RI are recognized: inflammatory CAA (ICAA), presenting with a perivascular pattern of inflammation, and amyloid-beta-related angiitis (ABRA), which demonstrates a more destructive, transmural inflammatory infiltrate [5].

CAA typically occurs in older individuals, with prevalence estimates ranging between 30 and 40 cases per 100,000 people, and is most frequently diagnosed in individuals between the seventh and eighth decades of life [4]. CAA-RI most commonly presents with chronic or subacute cognitive decline, reported in approximately 50%-70% of cases. Other frequent clinical manifestations include seizures (∼35%), headaches (∼30%), and a variety of focal neurological deficits. Acute presentations occur in about 10%-20% of cases [6,7]. Early recognition and timely immunosuppressive treatment can result in favorable clinical outcomes, including reversibility of symptoms [8].

Diagnosing CAA-RI can be particularly challenging due to its clinical similarities with other neurological disorders, such as ischemic stroke and brain tumors, often resulting in delays in accurate diagnosis or even initial misdiagnosis as a stroke mimic. Although brain biopsy remains the definitive diagnostic standard, it is invasive and carries inherent risks. Thus, diagnostic criteria developed by Auriel et al., which integrate clinical and radiological characteristics, have gained widespread acceptance for diagnosis [9].

While most patients improve significantly with corticosteroid treatment and typically experience only one episode, there have been infrequent case reports of recurrent episodes, with approximately one out of four patients experiencing relapses years after their initial presentation [10]. The literature on CAA-RI predominantly consists of isolated case reports or small case series, underscoring the rarity of this condition.

We present the case of a 63-year-old female with CAA-RI, who experienced recurrent stroke-like presentations seven years apart. We aim to discuss the diagnostic dilemma posed by repeated stroke-like presentations and to highlight the difficulties faced by clinicians, the importance of pattern recognition, and the need to maintain a high index of suspicion for this rare yet important inflammatory condition.

## Case presentation

**Figure 1:**
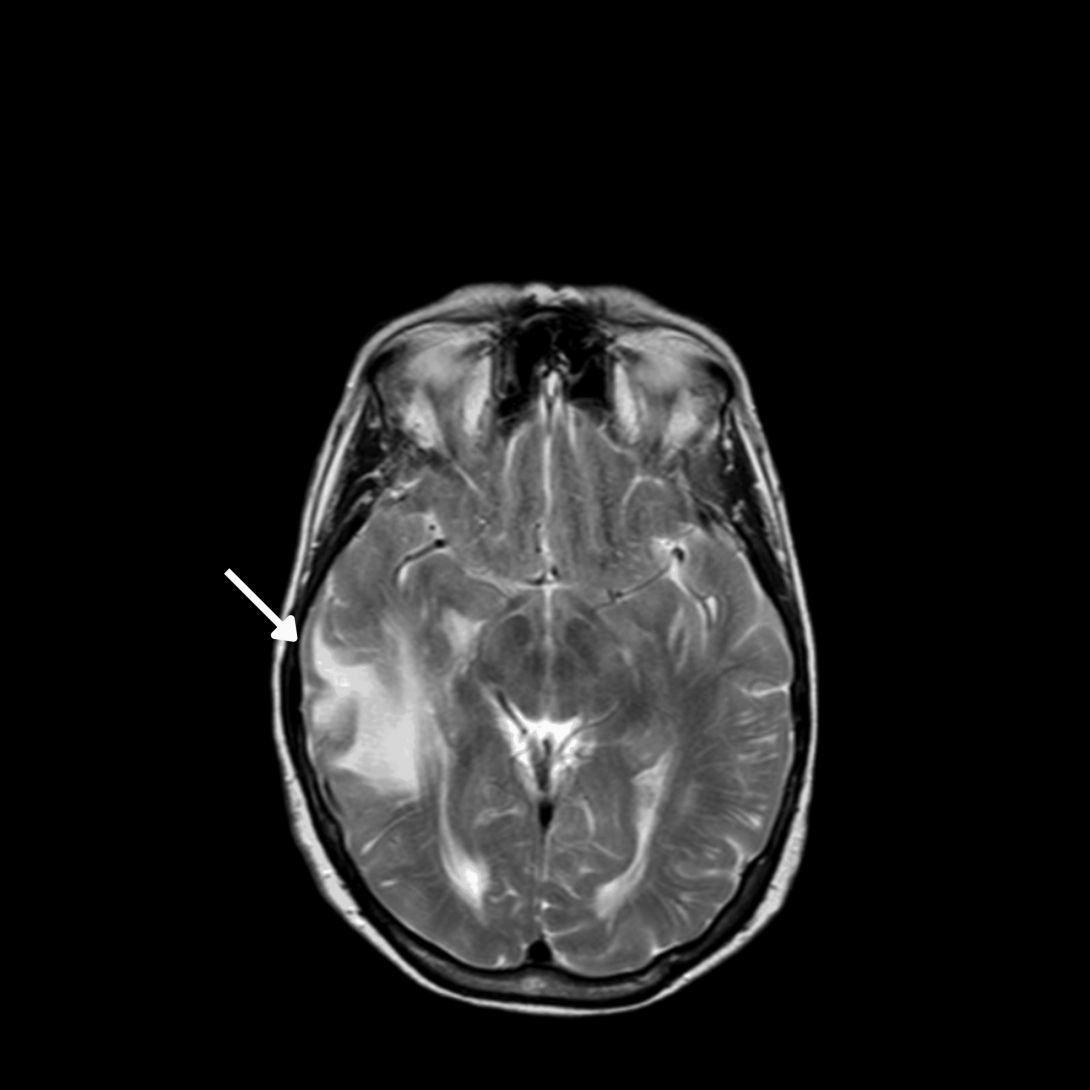
MRI performed in September 2013 showed increased T2 signal (arrow) in the right temporal lobe. MRI, magnetic resonance imaging

A 63-year-old female presented in 2013 with an unwitnessed fall secondary to collapse. She was able to walk back home with the aid of a passerby. Her daughter also noted intermittent disorientation over the next two days. She then developed an acute onset of vertigo, requiring urgent hospital admission as a suspected stroke. On arrival, she was alert with a Glasgow Coma Scale (GCS) score of 15/15 and was hemodynamically stable, with no focal neurological deficits on examination. CT of the head ruled out hemorrhage but showed extensive vasogenic oedema in the right temporal lobe. Further contrast-enhanced MRI of the head revealed confluent cortical FLAIR hyperintensity, along with hyperintensity on DWI and ADC sequences in the right temporal lobe (Figure 1). The initial differential included low-grade glioma and ischemic stroke. She was initiated on dexamethasone 8 mg twice a day in view of the possibility of glioma. Her MRI images were discussed in the nerve radiology MDT. Interestingly, it was noted that the gradient-echo T2-weighted axial images showed punctate areas of hemosiderin deposition in both cerebral hemispheres. The consensus was that there was no pathological enhancement following contrast, and given the involvement of multi-focal areas, the appearances were not typical for gliomatosis cerebri or metastatic disease. Based on clinical and radiological findings, she was diagnosed with amyloid angiopathy/vasculopathy. As she was clinically responding well, dexamethasone was continued at 8 mg twice daily, followed by a weaning regimen with a dose reduction of 2 mg every three days.

**Figure 2:**
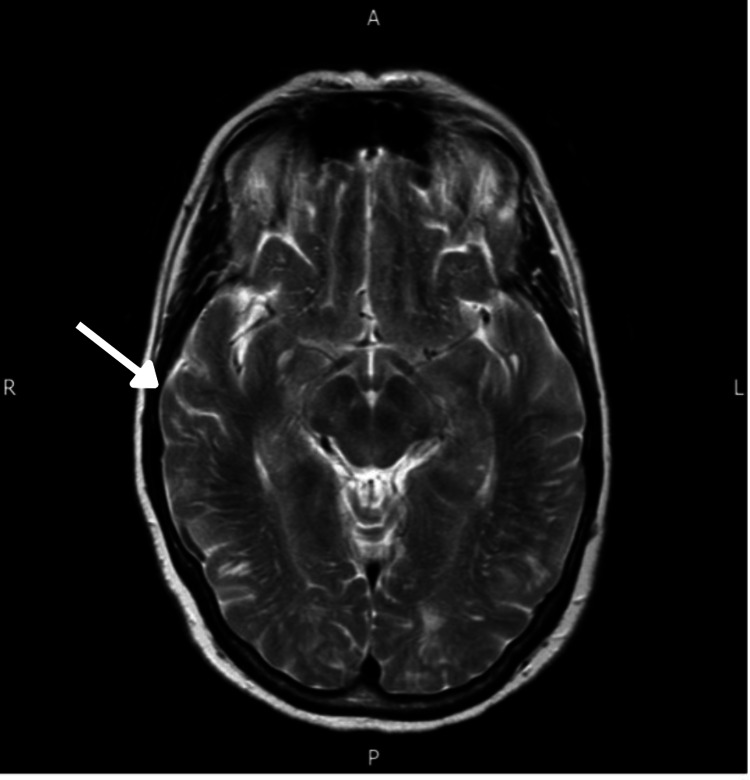
MRI performed in October 2013 showed complete resolution of the hyperintensities (arrow). MRI, magnetic resonance imaging

Surveillance MRI of the head with contrast (Figure 2), performed two months later, showed complete radiological resolution, i.e., resolution of edema in the right frontotemporal regions and unremarkable intracranial appearances with no abnormal post-contrast enhancement.

**Figure 3:**
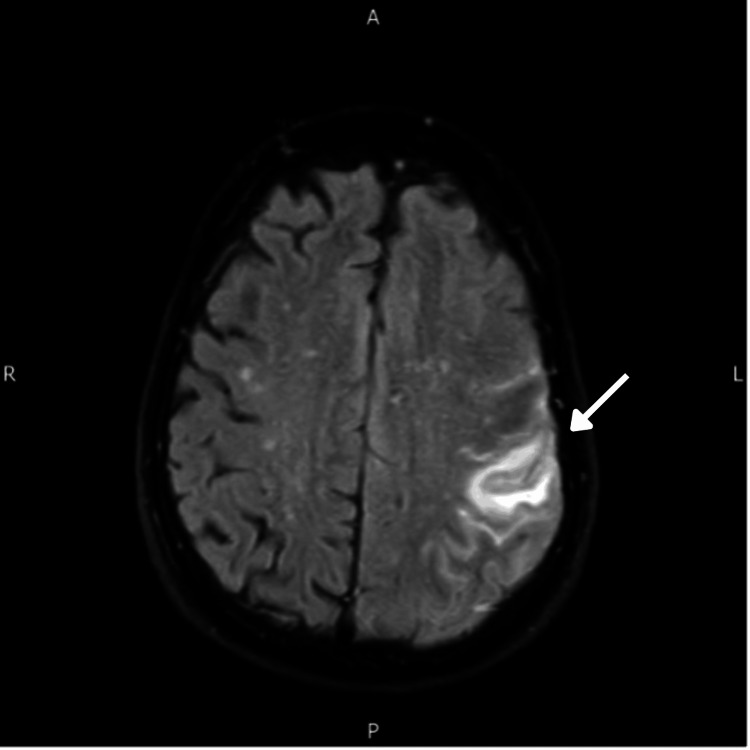
MRI April 2020 showing a new increase in T2 and FLAIR signal in the left temporoparietal lobe (arrow). MRI, magnetic resonance imaging; FLAIR, fluid-attenuated inversion recovery

**Figure 4:**
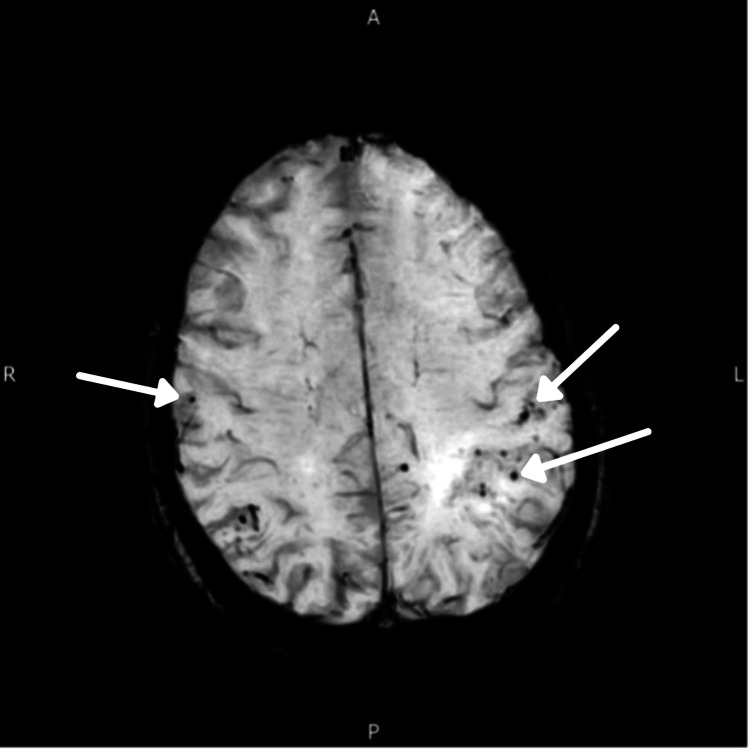
MRI June 2020 was consistent with a CAA pattern (microbleeds; arrows). CAA, cerebral amyloid angiopathy; MRI, magnetic resonance imaging

In April 2020, she again presented to the hospital as a stroke alert, with complaints of bilateral arm numbness and aphasia lasting for one hour. Her initial National Institutes of Health Stroke Scale (NIHSS) score was 4, and she was noted to be confused, with an Abbreviated Mental Test score of 5/10. Initial CT of the head showed new loss of parenchymal differentiation in the left MCA territory with subtle edema, highly suspicious for an evolving left MCA territory infarct. She was treated according to the stroke protocol with high-dose aspirin, as she was outside the thrombolysis window. MRI of the head (Figure 3) was performed as part of the diagnostic workup for stroke and initially suggested a subacute left MCA territory infarct. In view of her previous history of intermittent confusion and the MRI findings, her case was discussed at the neuroradiology MDT. Differential diagnoses included focal meningitis and a space-occupying lesion, for which contrast-enhanced MRI and lumbar puncture (LP) were subsequently performed. The MRI head with contrast (Figure 4) showed subcortical T2 hyperintensity in the left temporoparietal region, with no underlying mass effect and no enhancement on post-gadolinium T1-weighted or FLAIR images. This scan was re-discussed at the stroke radiology MDT, and the left posterior frontal changes were considered to represent epileptogenic cortical changes. There was also a subarachnoid FLAIR high signal, raising the possibility of cerebritis or meningitis, as well as potential early cerebral amyloid changes. Lumbar puncture was inconclusive for meningitis. She subsequently underwent an EEG, which showed findings consistent with recent-onset bilateral (left > right) posterior cerebral dysfunction that was epileptogenic, further supporting the possibility of seizures. She was discharged with a diagnosis of CAA-related seizure disorder. The plan was to complete a course of aspirin, continue levetiracetam, which had been started earlier during the admission, and repeat the MRI brain in two months.

**Figure 5:**
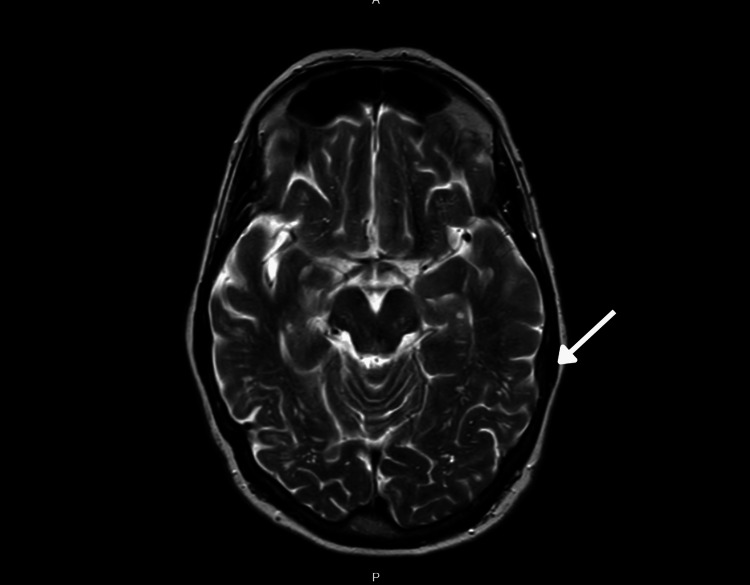
MRI head with contrast done in June 2021 showed resolution of diffuse subcortical white matter changes in the left temporoparietal region. MRI, magnetic resonance imaging

Her repeat MRI head with contrast after two months showed significant resolution of cortical T2 and FLAIR hyperintensity in the left temporoparietal region. A subsequent MRI with contrast a year later (Figure 5) showed near-complete resolution of the diffuse subcortical white matter changes in the same region.

## Discussion

CAA-RIIn 1909, a German scientist named Gustav Oppenheim published a paper that first described amyloid deposition in the central nervous system (CNS). Twenty-nine years later, in 1938, W. Z. Scholz published the first paper describing what would later be known as CAA [11].

Pathophysiology and presentation

CAA is characterized by amyloid beta-peptide deposits within small- to medium-sized blood vessels of the brain and leptomeninges. It is an important cause of lobar intracerebral hemorrhage in older adults. In addition to intracerebral hemorrhage, CAA may present with transient neurological symptoms, an inflammatory leukoencephalopathy, as a contributor to cognitive impairment, or with incidental microbleeds or hemosiderosis on MRI [12,13]. CAA associated with inflammation is an increasingly recognized condition, characterized by an inflammatory response to the vascular deposits of β-amyloid within the brain blood vessels [14]. CAA-RI presents with symptoms of acute or sub-acute cognitive decline, seizures, headaches, and focal neurological deficits. The exact mechanism of the disease process remains unclear, but the most widely accepted hypothesis is that Aβ deposition triggers an inflammatory response [15].

CAA-RI is more prevalent in the elderly population, but there is no specific trend or data to suggest any gender predisposition based on our literature review.

Diagnostic challenges

MRI brain is the most important imaging modality, and findings supportive of CAA-RI include patchy or confluent T2 hyperintensity of subcortical white matter lesions, which are mostly asymmetric, in addition to the presence of multiple, strictly lobar cerebral microbleeds (CMBs) and cortical superficial siderosis (cSS) on T2∗ or susceptibility-weighted imaging (SWI) [16]. Blood tests may reveal raised inflammatory markers, and cerebrospinal fluid (CSF) may reveal increased protein and anti-Aβ autoantibodies [17], but in our patient, they were normal. apolipoprotein E (APOE) ε4 allele is currently the only confirmed risk factor for CAA-RI [18]. Definitive diagnosis of CAA-RI requires typical clinical manifestation, typical MRI features, and brain biopsy [19].

The challenges encountered in diagnosing CAA-RI were also seen in our patient, who had repeated presentations with varied clinical symptoms each time. Initially, she presented with confusion and vertigo following a recent, unwitnessed fall. MRI head showed increased T2 and FLAIR signal, along with hyperintensity on DWI and ADC in the right temporal lobe. Glioma and stroke were considered as possible differentials. MDT discussion helped support an early diagnosis of probable amyloid angiopathy based on clinical symptoms and radiological review. The lack of generalized patchy meningeal enhancement, along with the presence of punctate hemosiderin deposits suggestive of microbleeds, made primary glioma an unlikely diagnosis and amyloid angiopathy more probable. This was further supported by the clinical response to steroids and complete radiological resolution on repeat MRI. High-dose steroids are the mainstay of treatment. From our literature review, IV methylprednisolone followed by oral prednisolone has been used for CAA-RI. In our patient, however, dexamethasone, which was initially started due to suspicion of glioma, was continued, as the patient was showing a clinical response. Her second presentation to the hospital, seven years later, was with bilateral arm paresthesia and aphasia, which resolved within an hour. CT head was suggestive of a left middle cerebral artery stroke, which was confirmed on further MRI, and she was started on high-dose aspirin. A prompt decision was made to hold an MDT discussion, taking her previous history of confusion and varied radiological imaging changes into account. The differentials at this point were meningitis and a space-occupying lesion, which were ruled out: lumbar puncture was inconclusive for meningitis, and contrast MRI showed changes consistent with elliptical cortical abnormalities, which were further confirmed on EEG. However, it is difficult to ascertain whether these changes were a sequel or complication of her first presentation, or whether they represented new amyloid deposition on the left side secondary to ongoing microangiopathic changes over the seven-year period.

CAA-RI is largely a diagnosis of exclusion, meaning that other possible causes should be ruled out before diagnosing CAA-RI based on the modified probable CAA-RI criteria.

Definitive diagnosis of CAA-RI requires typical clinical manifestations, characteristic MRI features, and brain biopsy [19]. Considering the risks associated with brain biopsy, Auriel et al. validated the modified probable CAA-RI criteria, which have good sensitivity and excellent specificity, and can be incorporated into clinical practice to identify patients.

Considering the risk of brain biopsy, the modified probable CAA-RI criteria, which have good sensitivity and excellent specificity, validated by Auriel et al., can be incorporated into clinical practice for identifying patients. According to the criteria [9], a diagnosis of probable CAA-RI was made in our patient based on these five aspects: Clinical criteria - the patient was over 40 years old, presented with headache, and had no acute intracerebral hemorrhage (ICH). Laboratory and imaging results showed no evidence of neoplastic, infectious, or other causes. Radiological criteria - MRI showed asymmetric cortico-subcortical white matter hyperintensity (WMH) lesions, with no history of prior ICH.

Treatment

Although the mainstay of treatment for CAA-RI is high-dose corticosteroids, with or without immunotherapy [10], spontaneous improvement in symptoms and resolution of imaging abnormalities have been reported [20], which was also observed in our patient on surveillance MRI scans following both presentations. In the first presentation, the patient received a short course of dexamethasone, with no further steroids. In the second presentation, spontaneous recovery was observed. Hence, it is important to tailor treatment to the individual based on their clinical presentation and response to therapy.

In summary, we report a woman who presented to the hospital twice as a stroke call with focal neurological deficits. Subsequent imaging initially suggested an infarct or a space-occupying lesion, which appeared to have *vanished* on follow-up surveillance scans. The consensus after both MDT discussions was that her symptoms were related to CAA-RI.

The aim is to highlight CAA-RI as an important differential for T2 hyperintensities on MRI, within the clinical context of a patient presenting with repeated stroke-like episodes and the associated MRI findings, as discussed. CAA-RI is an uncommon condition; therefore, a high degree of clinical suspicion is required to make the diagnosis.

## Conclusions

CAA-RI is a relatively rare but increasingly more understood condition. Currently, we face challenges in establishing its diagnosis, determining appropriate treatment, and creating a robust follow-up plan. We should maintain a high index of suspicion for CAA-RI in patients presenting with progressive cognitive decline, seizures, headaches, or focal deficits, especially when the MRI brain shows T2 or FLAIR hyperintensities along with CMBs or cSS. Prompt diagnosis and management can positively affect the outcome of the disease, as it is potentially reversible.

However, because there is a lot of variation in terms of presentation and subsequent radiological images, this diagnostic challenge remains high and warrants a high index of suspicion.
